# Segmental vitiligo of the lid margin with leukotrichia of lashes in a child

**DOI:** 10.5935/0004-2749.2023-0091

**Published:** 2024-06-20

**Authors:** Janani Rajagopal

**Affiliations:** 1 Department of Ophthalmology, Jawaharlal Institute of Postgraduate Medical Education and Research (JIPMER), Karaikal, Puducherry, India

**Figure 1:**
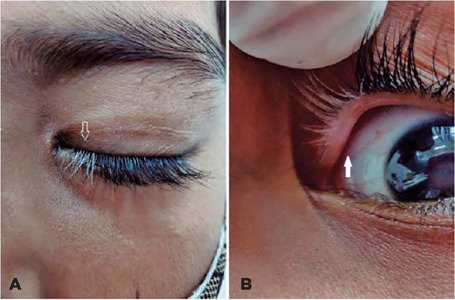
External photograph of the medial part of the upper lid of the left eye: (A) Leukotrichia of the lashes (arrow). (B) Lid margin segment (underlying the gray lashes) with segmental vitiligo (solid arrow) without the involvement of the overlying lid skin.

A 5-year-old child was brought by the parents with complaints of graying of lashes. Poliosis of the left medial upper lid lashes (Figure 1A) and segmental vitiligo of the underlying lid margin (Figure 1B) were noted on examination. No signs of uveitis or infection were seen. No other areas of hypopigmentation were observed. The child was diagnosed with “Segmental vitiligo with leukotrichia”. After appropriate laboratory workups and referrals, tacrolimus 0.1% ointment was started for 6 weeks, and the child is currently under follow-up. Vitiligo is an autoimmune disease that progressively destroys skin melanocytes^(1)^ and secondarily involves the hair follicles, which causes leukotrichia. Follicular vitiligo of the lashes is a differential diagnosis that primarily affects the melanocytic reservoir of the hair follicle^(2)^ inducing primary depigmentation of the follicle without initial involvement of the surrounding skin. Topical calcineurin inhibitors, Janus Kinase inhibitors, steroids, and surgical modalities such as follicular unit extraction have been attempted with limited success.
